# CD133^+^ Anaplastic Thyroid Cancer Cells Initiate Tumors in Immunodeficient Mice and Are Regulated by Thyrotropin

**DOI:** 10.1371/journal.pone.0005395

**Published:** 2009-04-30

**Authors:** Susan Friedman, Min Lu, Atara Schultz, Dolly Thomas, Reigh-Yi Lin

**Affiliations:** 1 Department of Medicine, Mount Sinai School of Medicine, New York, New York, United States of America; 2 Department of Developmental and Regenerative Biology, Mount Sinai School of Medicine, New York, New York, United States of America; 3 Department of Gene and Cell Medicine, Mount Sinai School of Medicine, New York, New York, United States of America; 4 The Black Family Stem Cell Institute, Mount Sinai School of Medicine, New York, New York, United States of America; INSERM, France

## Abstract

**Background:**

Anaplastic thyroid cancer (ATC) is one of the most lethal human malignancies. Its rapid onset and resistance to conventional therapeutics contribute to a mean survival of six months after diagnosis and make the identification of thyroid-cancer-initiating cells increasingly important.

**Methodology/Principal Findings:**

In prior studies of ATC cell lines, CD133^+^ cells exhibited stem-cell-like features such as high proliferation, self-renewal and colony-forming ability *in vitro*. Here we show that transplantation of CD133^+^ cells, but not CD133^−^ cells, into immunodeficient NOD/SCID mice is sufficient to induce growth of tumors *in vivo*. We also describe how the proportion of ATC cells that are CD133^+^ increases dramatically over three months of culture, from 7% to more than 80% of the total. This CD133^+^ cell pool can be further separated by flow cytometry into two distinct populations: CD133^+/high^ and CD133^+/low^. Although both subsets are capable of long-term tumorigenesis, the rapidly proliferating CD133^+/high^ cells are by far the most efficient. They also express high levels of the stem cell antigen Oct4 and the receptor for thyroid stimulating hormone, TSHR. Treating ATC cells with TSH causes a three-fold increase in the numbers of CD133^+^ cells and elicits a dose-dependent up-regulation of the expression of *TSHR* and *Oct4* in these cells. More importantly, immunohistochemical analysis of tissue specimens from ATC patients indicates that CD133 is highly expressed on tumor cells but not on neighboring normal thyroid cells.

**Conclusions/Significance:**

To our knowledge, this is the first report indicating that CD133^+^ ATC cells are solely responsible for tumor growth in immunodeficient mice. Our data also give a unique insight into the regulation of CD133 by TSH. These highly tumorigenic CD133^+^ cells and the activated TSH signaling pathway may be useful targets for future ATC therapies.

## Introduction

Thyroid cancer is the most common type of endocrine cancer [1]. Its incidence is increasing more rapidly than any other solid tumor - about 3 percent per 100,000 people each year [2] - and it is now the seventh most common cancer in women [1]. More than 90% of thyroid cancers are derived from thyroid follicular cells, are well differentiated and have a favorable prognosis. In contrast, anaplastic thyroid cancer (ATC), an undifferentiated thyroid cancer, is significantly more severe than other thyroid cancers and has a poor prognosis. Ninety percent of patients with ATC die within six months. Although ATC accounts for more than 50% of deaths associated with thyroid cancer every year, the causes of this disease are largely unknown. Current treatments for ATC are aggressive - including surgery, radiation therapy and chemotherapy – but no study has shown a convincing improvement in survival [3], perhaps because they do not adequately target the cancer-initiating cells. Most cancer therapies target differentiated or differentiating cells, regardless of whether or not they are cancerous. However, if the disease is due to cancer stem cells (CSCs) [4], [5], this could be the wrong approach. Like normal stem cells, CSCs can both self-renew and produce differentiated progeny, including a phenotypically diverse tumor cell population to drive tumorigenesis. Several lines of evidence suggest that CSCs, which are highly resistant to standard chemotherapeutic agents and radiation, sustain the disease in late phases of malignancy. To date, CSCs have been isolated based on their ability to express specific cell surface molecules in hematologic malignancies and epithelial-cell-derived cancers, including acute myeloid leukemia (CD34^+^CD38^−^CD123^+^) [5], mammary carcinoma (CD44^+^CD24^low^) [6], brain tumors (CD133^+^) [7], colon cancer and melanoma (CD133^+^) [8]–[11].

CD133 (prominin-1) is a five-transmembrane domain glycoprotein specifically expressed on populations of hematopoietic stem and progenitor cells from fetal and adult cord blood, peripheral blood and bone marrow [12]–[15]. Although its biological function remains unknown, it also serves as a marker of stem cells in a variety of non-hematopoietic tissues, including neural and glial cells in the fetal brain as well as prostatic epithelia, muscle, kidney, liver and corneal stroma, and some cancerous tissues [15]–[24]. Recently, Zito *et al* reported that, of four human ATC cell lines examined, two — ARO and KAT-4 — contain subpopulations of CD133^+^ cells that exhibit stem cell-like features such as rapid proliferation, an ability to self-renew and form colonies, and resistance to chemotherapy-induced apoptosis *in vitro* [25]. As a result, these populations are believed to be able to initiate tumor growth, although this hypothesis has not yet been validated in animal models.

Here we evaluate the tumorigenic potentials of ATC-derived CD133^+^ populations *in vivo*. We used fluorescent-activated cell sorting (FACS) to further divide the CD133^+^ cells into two distinct fractions: CD133^+/high^ and CD133^+/low^. We then compared the ability of CD133^+/high^, CD133^+/low^ and CD133^−^ cells to engraft and give rise to subcutaneous tumors in NOD/SCID mice. We used two xenotransplantation models in our study to explore the possible existence of CSCs in ATC: limiting dilution transplantation experiments to determine whether our xenograft system was qualitative and capable of detecting single tumor-initiating cells, and a serial xenograft model to test the cells' self-renewal and lineage capacities. We observed that, although both CD133^+/high^ and CD133^+/low^ cells generate subcutaneous xenograft tumors that display histology resembling the primary tumors, CD133^+/high^ cells initiate earlier and more aggressive tumor growth. We also found that thyroid-stimulating hormone (TSH), the main regulator of thyroid gland function, is important for the growth of the CD133^+^ population. Unlike normal thyroid cells, ATC tissue specimens were clearly positive for CD133. Our findings, together with the identification of thyroid CSCs and the elucidation of the role of TSH in CD133^+^ cell growth, may lead to new diagnostic markers and therapies to treat this devastating disease.

## Results

### CD133 is expressed on human ATC cell lines but not on papillary thyroid cancer cell lines

**Figure 1:**
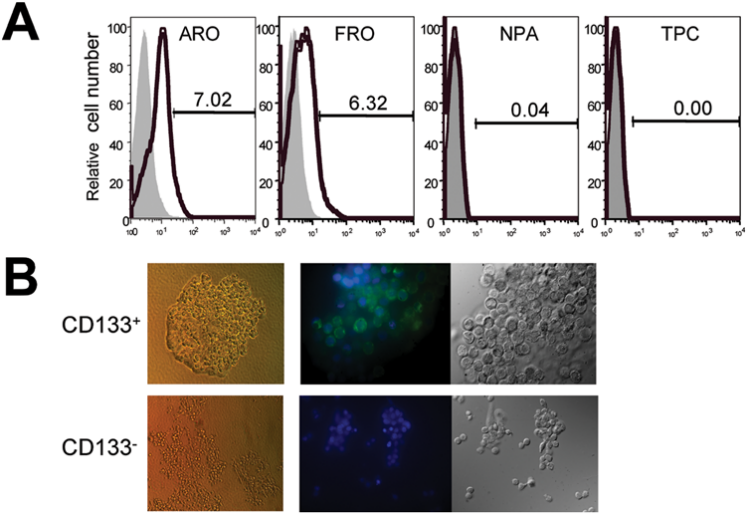
A CD133^+^ population of cancer cells is present in human ATC cell lines. (A) Flow cytometric analysis of CD133 expression in ARO, FRO, NPA and TPC cell lines. Black lines represent positive staining for CD133, grey shows isotype controls. (B) Sorted CD133^+^ cells form tight round colonies in two-dimensional cell cultures, while CD133^−^ cells grow independently as small round cells. Immunofluorescent images of CD133^+^ cells (upper middle panel; 63× magnification) show CD133 expression as a *green signal* at the cell surface. In contrast, CD133^−^ cells lack this signal (lower middle panel; 20× magnification).

To determine whether a CSC population exists in ATC, we examined CD133 expression in a panel of human thyroid cancer cell lines. Flow cytometric analysis revealed that CD133 is expressed in two ATC cell lines, ARO (in which 7.02% of cells are CD133^+^) and FRO (in which 6.32% of cells are CD133^+^), but not in either of two well-differentiated papillary thyroid cancer cell lines (NPA and TPC) (Fig. 1A). These results suggest that CD133 expression is uniquely associated with undifferentiated ATC. Immunofluorescent staining confirms the expression of CD133 on the surface of CD133^+^ but not CD133^−^ cells (Fig. 1B). Moreover, the two CD133 populations grow differently in culture. In tumors, CD133^+^ cells exhibit pleomorphic giant cell nuclei and appear granular and dense; in two-dimensional cultures they form tight round colonies. In contrast, CD133^−^ cells are devoid of most of these features. Similar to ARO cells, they grow independently as small, round cells in culture (Fig 1B).

### CD133^+^ cells express high levels of the genes for Oct4 and TSHR

**Figure 2:**
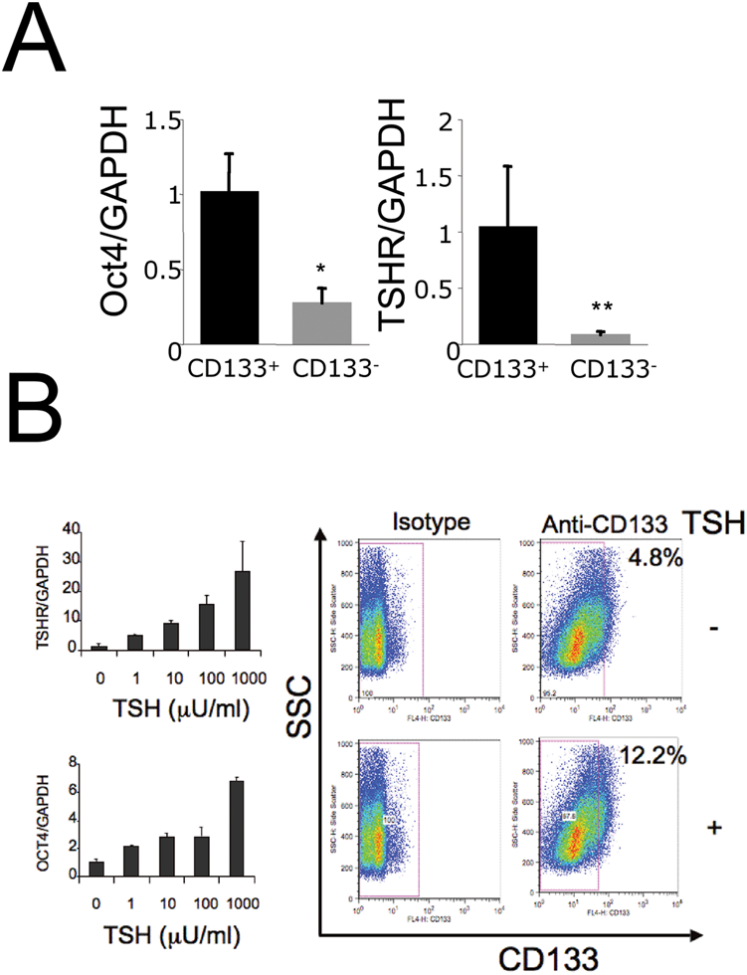
CD133^+^ cells express high levels of *Oct4* and *TSHR* genes. (A) qRT-PCR analysis indicates that CD133^+^ cells express higher levels of *Oct-4* and *TSHR* than do CD133^−^ cells. Results are shown as mean and s.e.m. Asterisk: *P*<0.05; two asterisks: *P*<0.01. (B) TSH upregulates the expression of *TSHR* and *Oct4* and increases the number of CD133^+^ cells. Left panel: human recombinant TSH elicits a dose-dependent upregulation of *TSHR* and *Oct4* mRNA levels in unsorted ARO cells. Right panel: the number of CD133^+^ cells increases three-fold in response to TSH stimulation.

Quantitative real-time polymerase chain reaction (qRT-PCR) analysis indicated that CD133^+^ cells express about four times as much *Oct-4* as do CD133^−^ cells (Fig. 2A; 1.02±0.25 (mean±s.e.m.) *versus* 0.27±0.10 (mean±s.e.m.), *P*<0.05). Oct4 is a member of the POU family of transcription factors and plays a key role in the maintenance of pluripotency and the proliferation of embryonic stem cells. The expression of Oct4 in CD133^+^ cells indicates that, consistent with a previous report [25], this population has stem-cell-like properties that are not shared with CD133^−^ cells. The CD133^+^ population also expresses approximately 13-fold higher levels of *TSHR* than does the CD133^−^ population (Fig. 2A; 1.05±0.53 (mean±s.e.m.) *versus* 0.08±0.03 (mean±s.e.m.), *P*<0.01). TSHR is a G protein-coupled glycoprotein receptor that stimulates the growth of normal and cancerous thyroid cells in the presence of TSH. Our discovery that CD133^+^ cells express significantly higher levels of TSHR implies that TSH also regulates the growth of CD133^+^ cells.

### Enhanced cell proliferation and up-regulation of Oct4 and TSHR genes in response to TSH signaling

Clinical studies have indicated that elevated TSH levels may be a marker for the development of thyroid cancer [26]–[30]. In particular, most thyroid cancer patients have above-normal TSH levels. Because CD133^+^ cells express much higher levels of TSHR than do CD133^−^ cells, we examined the effect of TSH on CD133^+^ populations. ARO cells cultured with 0–1000 µU/ml recombinant human TSH for 48 hours exhibited a dose-dependent increase in the relative expression of both the *TSHR* and *Oct4* genes (Fig. 2B). The number of ARO cells expressing CD133 also increased about three-fold in response to TSH treatment (Fig. 2B). Together, these observations demonstrate that TSH induces the proliferation of the CD133^+^ populations *in vitro*.

### ARO cells reconstitute human ATC tumors in vivo

**Figure 3:**
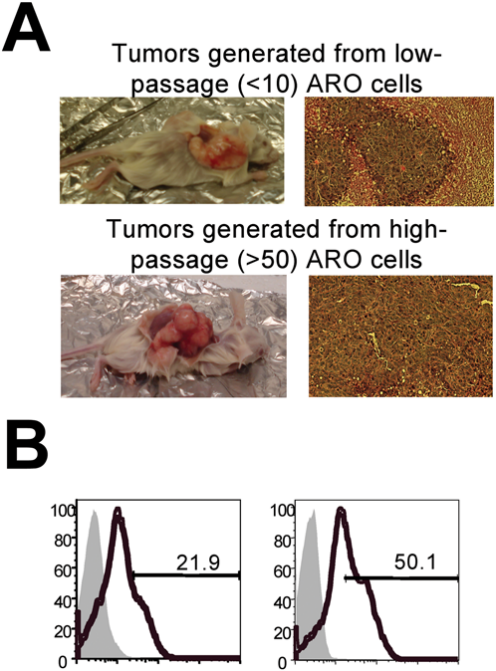
ARO cells reconstitute human ATC tumors in NOD/SCID mice and high-passage ARO cells generate faster-growing, larger tumors in NOD/SCID mice than do low-passage ARO cells. (A) All mice injected with 10^6^ ARO cells developed tumors within 21 days. Hematoxylin-eosin analysis of these xenografts showed that they are remarkably similar to tumor sections from human patients. Note that tumors generated from high-passage ARO cells formed larger and more aggressive tumors when subcutaneously transplanted into NOD/SCID mice than did tumors generated from low-passage ARO cells. (B) FACS analysis show that ARO cells of high passage number (right panel, passage number>50) contain a greater proportion of CD133^+^ cells than do ARO cells of low passage number (left panel, passage number<10).

**Figure 4:**
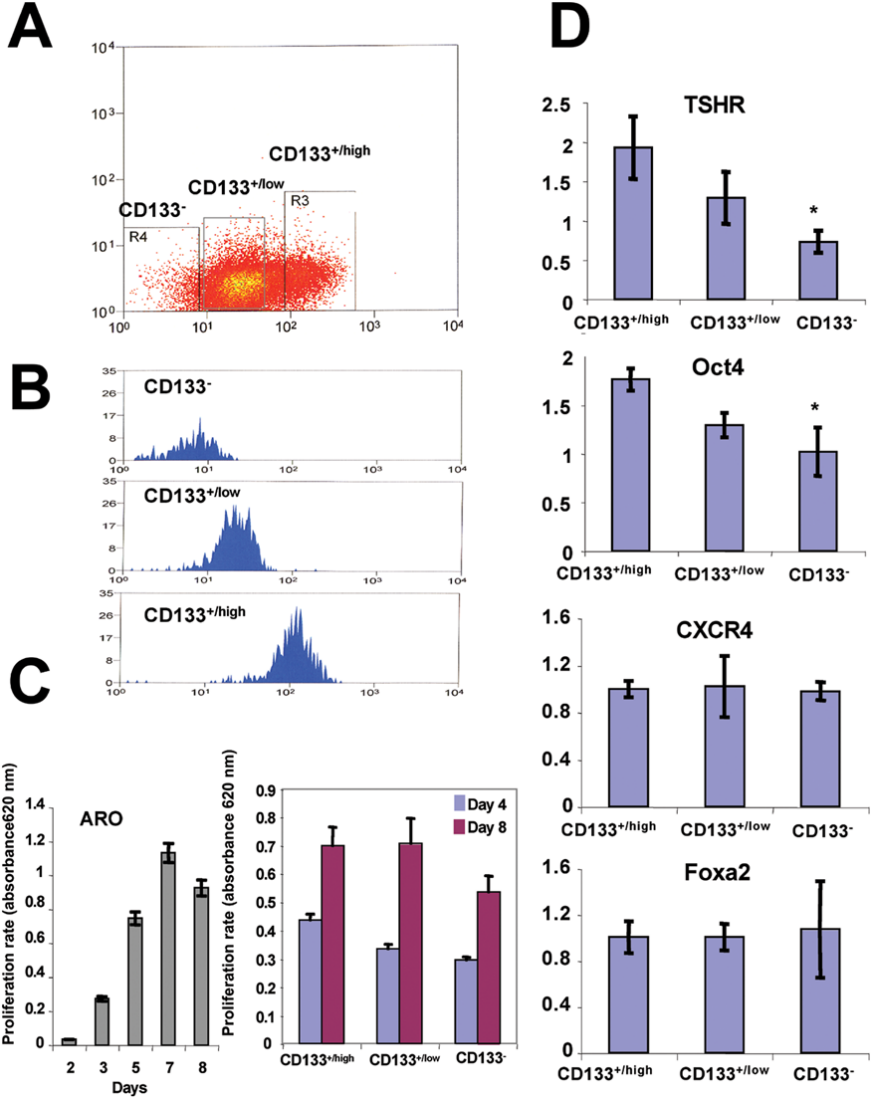
CD133^+/high^ subpopulations express higher levels of *TSHR* and *OCT4* genes than do CD133^+/low^ and CD133^−^ subpopulations. (A) The CD133^+^ population can be further separated into CD133^+/high^ and CD33^+/low^ populations by FACS. (B) The purity of each sorted fraction was confirmed by FACS analysis. (C) ARO cell proliferation assay (left panel). Cell proliferation assays indicate that CD133^+/high^ and CD133^+/low^ cells proliferate more rapidly than CD133^−^ cells at culture days four and eight (right panel). (D) qRT-PCR analysis. Results are shown as mean and s.e.m. Asterisk, *P*<0.05.

To develop a reliable model of human ATC biology, we established four ARO subcutaneous xenografts in eight-week-old female NOD/SCID mice. Mice injected with single-cell suspensions of 10^6^ ARO cells developed palpable tumors within a few days and visible tumors within 21 days. These tumors are remarkably similar to those from human patients and exhibit little resemblance to the original thyroid follicle structure (Fig. 3A). Importantly, we found that the tumorigenic potential of the ARO cells varies with their passage number: cells that had been cultured longer formed larger and more aggressive tumors when transplanted into NOD/SCID mice than did cells of lower passage number (Fig. 3A). We speculate that this phenomenon is due to the positive selection over time of the more robustly proliferating CD133^+^ cells at the expense of the CD133^−^ cells. Indeed, we found that two ARO cultures that had been passaged 50 or more times contained a higher proportion (∼50.1%) of CD133^+^ cells than did ARO cultures of lower passage numbers (∼21.9%)(Fig. 3B). Together, these results demonstrate that the human ARO cell line contains cells with the capacity to form new tumors in NOD/SCID mice and that CD133 expression levels are positively correlated with tumor formation. Furthermore, the rapid proliferation of CD133^+^ cells results in the robust positive selection of these cells over time—from the original 7.02% (Fig. 1A) of the total population to more than 80% after three months of continuous culture (Fig. 4). These results demonstrate that an extended period of culture can transform CD133^−^-dominant populations into CD133^+^-dominant populations.

### CD133^+/high^ cells express higher levels of TSHR and Oct4 genes than do CD133^+/low^ and CD133^−^ cells

The CD133^+^ cell pool can be further separated by FACS into two distinct subpopulations: CD133^+/high^ and CD133^+/low^ (Fig. 4A). In a highly passaged culture of ARO cells, about 80% of the cells were CD133^+/low^, 16% were CD133^+/high^, and 4% were CD133^−^ (Fig. 4A). We confirmed the purity of the fractions after sorting (Fig. 4B) and conducted cell proliferation assays to determine whether there are any differences in the proliferation rates of these subpopulations. As expected, the CD133^+/high^ and CD133^+/low^ subpopulations proliferate more rapidly than the CD133^−^ subpopulation *in vitro* (Fig. 4C). qRT-PCR analysis further revealed that the CD133^+/high^ subpopulation expresses the highest levels of *Oct4* and *TSHR*, while CD133^−^ cells express the lowest levels of these genes (Fig. 4D). We also assessed the expression of *CXCR4*, the receptor for the stromal cell-derived factor-1 CXCL2 chemokine that is expressed in a variety of solid tumors including ATC [31], [32]. We found that the expression levels of neither *CXCR4* nor the endoderm marker *Foxa2* differed significantly among the three subpopulations (Fig. 4D).

### CD133^+/high^ cells are highly tumorigenic in vivo

To test our hypothesis that only a small subpopulation of highly proliferative ARO cells is responsible for tumor formation, we transplanted groups of NOD/SCID mice with increasing numbers of CD133^+/high^, CD133^+/low^ and CD133^−^ cells - from an amount normally unable to initiate tumor growth (∼1,000 cells) to an amount that always initiates tumor growth (∼100,000 cells). As shown in Table 1, injection of 100,000 to 10,000 CD133^+/high^ cells gave rise to visible tumors in all mice (4/4) within four weeks and 1 of 2 mice injected with 1,000 CD133^+/high^ cells developed a tumor. In contrast, although injection of 100,000 CD133^+/low^ cells also gave rise to new tumors (4/4), only 1 out of 4 mice developed a tumor after an injection of 10,000 CD133^+/low^ cells, and no tumors were observed when mice were injected with 1,000 CD133^+/low^ cells. No tumors were observed in mice injected with any number of CD133^−^ cells. Notably, subcutaneous tumors derived from the injection of CD133^+/high^ ARO cells grew more quickly in NOD/SCID mice than did tumors derived from CD133^+/low^ cells: mice injected with 100,000 CD133^+/high^ cells developed 250 mm^3^ tumors within 16 days and 1000 mm^3^ tumors within 35 days whereas mice injected with 100,000 CD133^+/low^ cells required 35 days to develop 250 mm^3^ tumors and 45 days to develop 1000 mm^3^ tumors (data not shown). These observations suggest that, although both CD133^+/high^ and CD133^+/low^ cells can initiate tumors, CD133^+/high^ cells initiate tumor growth more efficiently and grow more quickly than CD133^+/low^ cells.

**Table 1:** Limiting-dilution transplantation analysis of human ARO cells.

Cell source	Cell dose	Number of primary mice with tumors/(total number injected)	Number of secondary mice with tumors/(total number injected)
CD133^+/high^	100,000	4/4	4/4
	10,000	4/4	4/4
	1,000	1/2	3/4
CD133^+/low^	100,000	4/4	4/4
	10,000	1/4	4/4
	1,000	0/4	1/4
CD133^−^	10,000	0/4	
	1,000	0/4	
Bulk	1,000,000	4/4	

### Long-term tumorigenic potential of CD133^+/high^ and CD33^+/low^ cells in NOD/SCID mice

**Figure 5:**
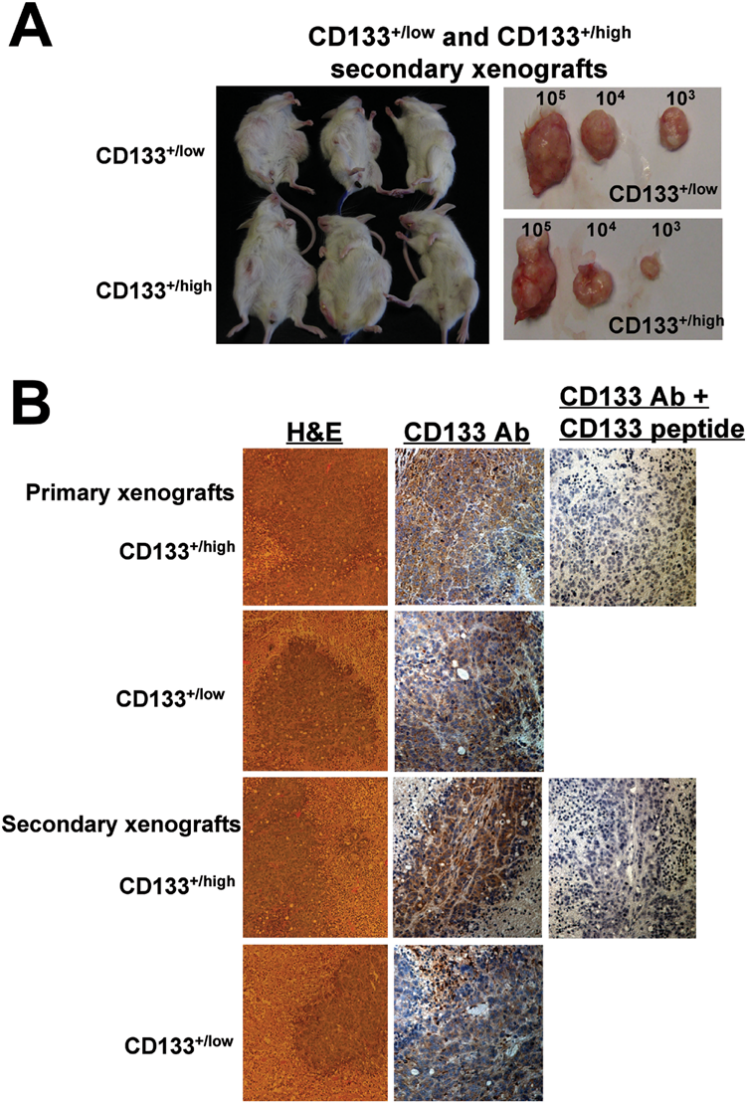
Long-term tumorigenic potential of CD133^+/high^ and CD133^+/low^ cells. (A) CD133^+/high^ and CD133^+/low^ subsets of ARO cells each exhibit long-term tumorigenic potential. Subcutaneous tumors in NOD/SCID mice derived from secondary injection of CD133^+/high^ and CD133^+/low^ cells. (B) Hematoxylin-eosin (H&E) and CD133 expression analysis of mouse xenografts generated from primary and secondary xenografts.

Cells from primary tumors originating from the injection of CD133^+/high^ and CD133^+/low^ cells were serially transplanted into NOD/SCID mice to determine the long-term tumorigenic potential of the cells. The limiting-dilution transplantation experiments described above indicate that the size of the tumors is positively correlated with the number of CD133^+/high^ and CD133^+/low^ cells injected. Tumors derived from CD133^+/high^ and CD133^+/low^ primary and secondary xenografts consistently reproduced the primary tumors at the histological level (Fig. 5). Although both subpopulations not only maintained their tumorigenic potential during *in vivo* passaging but also increased their aggressiveness, as indicated by the faster growth and increasing size of successively generated tumors, the CD133^+/high^ cells are overall more aggressive. Together these results suggest not only that CD133^+/high^ and CD133^+/low^ cells have long-term tumorigenic potential, but also that our xenograft model system quantitatively and qualitatively recapitulates tumorigenesis *in vivo*.

### CD133 is highly expressed in surgical samples of human ATC but not in normal thyroid cells

**Figure 6:**
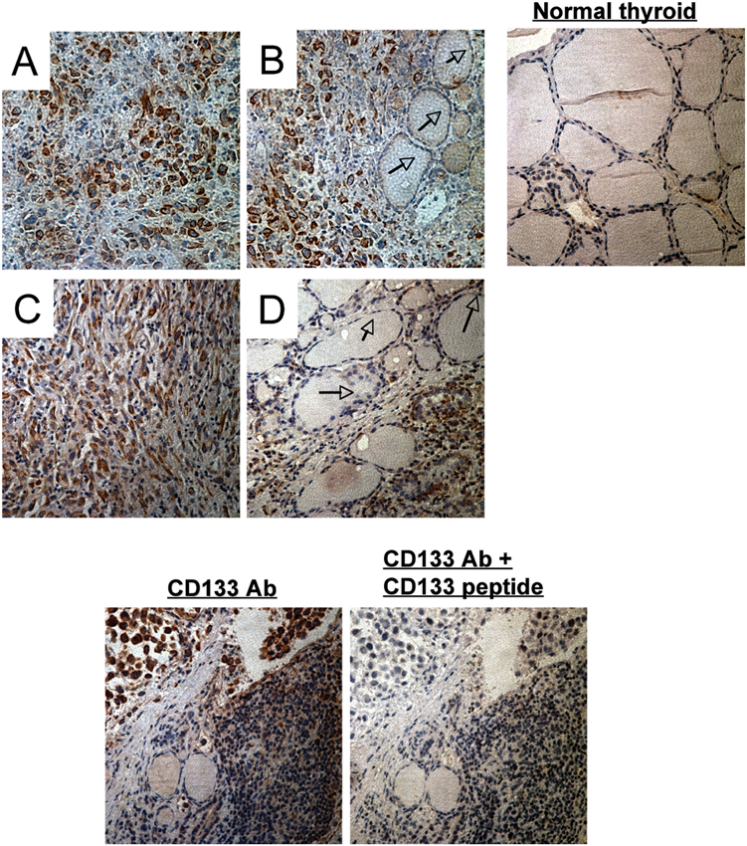
CD133 is highly expressed in surgical samples of human ATC but not in normal thyroid cells. (A–D) Immunohistochemical analysis of CD133 expression in ATC specimens. Positive cells are brown. Note that normal thyroid cells do not express CD133 (arrow). The photo on the right bottom is CD133 antibody plus peptide competition staining.

To understand the role of CD133 in human ATC, it is necessary to verify whether the CD133 protein is expressed in surgical samples derived from patients with pathological and clinical diagnoses of ATC. We used immunohistochemical staining to evaluate levels of CD133 expression in a set of archival, routinely processed, formalin-fixed, paraffin-embedded ATC patient tissue specimens (*n* = 10). We detected CD133 expression in 8 out of 10 (80%) ATC specimens. Staining intensity ranged from strong to moderate. Representative pictures of ATC specimens are shown in Fig. 6. Strikingly, CD133 is strongly expressed on tumor cells, but not on neighboring normal thyroid follicular cells (Fig. 6). Antibody specificity was demonstrated through peptide blocking. Together these data indicate that CD133 is overexpressed in ATC clinical samples with respect to normal thyroid tissues.

## Discussion

ATC is one of the most lethal of all human neoplasms. It is highly resistant to conventional therapies and its mortality rate approaches 100%. The relative rarity and rapidly fatal nature of ATC complicate both its diagnosis and treatment. The present study demonstrates for the first time that only a subpopulation of ATC cells is able to form tumors in NOD/SCID mice. We identified and isolated these cells based on the expression of a cell surface CSC marker, CD133, in the human ATC cell line ARO. Our data demonstrate that as few as 1,000 CD133^+/high^ cells and 10,000 CD133^+/low^ cells can form tumors in NOD/SCID mice. In contrast, mice injected with CD133^−^ cells remained tumor free throughout the study period. These data reveal that tumorigenic and non-tumorigenic cells co-exist within ATC, and imply that not all tumor cells are able to initiate neoplastic growth. Rather, anaplastic tumors are generated by a small subset of CSC cells that can self-renew and differentiate into the entire tumor population. Our *in vivo* data support a previously uncharacterized CSC model for ATC tumorigenesis [33], [34].

The current approach relies on two well-characterized human ATC cell lines: ARO and FRO. Zito *et al* previously reported that over 60% of ARO cells were CD133^+^, and that FRO cells did not express CD133 [25]. In contrast, we found that about 7% of both ARO and FRO cells were CD133^+^ (Fig. 1A). Furthermore, despite the fact that immunofluorescent staining by us (Fig. 1B) and others clearly show that CD133 is a cell surface protein, Zito's group described CD133 immunostaining as cytoplasmic and apical — atypical for a five-transmembrane glycoprotein [25]. There are several possible explanations for these discrepancies. First, because the sources of cell lines are different, it is possible that they inherited different characteristics. For example, we describe here how, like many CSCs, CD133^+/high^ cells proliferate more rapidly than CD133^−^ cells (Fig. 3). Over an extended period of continuous culturing, we observed that the proportion of CD133^+^ cells in the ARO culture increased dramatically from 7% to 80%. If Zito's group studied early-passage FRO cells they may have mistakenly concluded that the cells did not express CD133. A recent report by Schweppe *et al* also suggests that the purported ATC cell lines ARO and KAT-4 are actually derived from the colon cancer cell line HT-29 and are not of thyroid cancer origin [35]. Although it is not clear whether the cell lines used by Zito *et al* were contaminated, genetic analysis and DNA fingerprinting studies should be used to clarify the identity of these cell lines. Finally, it will be necessary to confirm our findings in fresh human ATC surgical tissue samples because cell lines do not always recapitulate all aspects of primary tumors. However, the rarity of the disease may make this difficult to achieve.

The rapid proliferation of CD133^+^ cells may at least partially explain ATC's fatal nature. Existing diagnostic methods are based upon several characteristics: a lack of radioactive iodine uptake, a direct extension into soft tissues, and a rapidly enlarging neck mass (mean size at presentation: 8 cm). Most patients are struggling with breathing problems by the time they are diagnosed, and most of these cancers are already stage IV—they have spread extensively to cervical lymph nodes and distant organs such as lung and bone, and are difficult to target and kill even with surgery. Because it is likely that the fast-growing nature of ATC and the subsequent high casualties are due to the highly proliferative and tumorigenic nature of CD133^+^ cells, it seems reasonable that future therapies should be designed to target CD133^+^ populations. Drug therapies targeting these populations are likely to differ from current chemotherapies for ATC patients and may be more successful in treating the disease.

Our immunohistochemical studies of clinical ATC specimens clearly showed that CD133 is expressed on tumor cells but not on neighboring normal thyroid cells. More importantly, tumors derived from CD133^+/high^ and CD133^+/low^ primary and secondary xenografts faithfully reproduced the primary tumors at the histological level in our xenograft models (Fig. 5). Together these results suggest that CD133^+^ cells maintain their tumorigenic potential during *in vivo* passaging and are indeed CSCs. CSCs self-renew and reconstitute their particular organ systems through a process of asymmetrical division that generates one new stem cell and one daughter cell capable of differentiation. They can also divide symmetrically to form either two normal daughter cells or two stem cells, depending on surrounding extracellular factors. Our data that CD133^+^ cells generate new tumorigenic CD133^+^ cells in addition to mixed populations of non-tumorigenic cells further support the notion that they are true CSCs.

Shmelkov *et al* recently reported that CD133 may not be a reliable marker for colon CSCs. Using transgenic mice expressing LacZ under the CD133 promoter, they found that CD133 was broadly expressed in normal and cancerous colonic epithelium [36]. Furthermore, they reported that although both CD133^+^ and CD133^−^ metastatic colon cancer cells can initiate tumors in NOD/SCID mice, CD133^+^ cells can give rise to the more aggressive CD133^−^ cells during the metastatic transition [36]. ATC is an undifferentiated thyroid cancer, and its behavior is largely different from colon cancer. For example, we found that normal thyroid cells do not express CD133. Moreover, both CD133^+/high^ and CD133^+/low^ subsets can initiate tumor growth in NOD/SCID mice, but CD133^+/high^ cells generate more aggressive tumors than CD133^+/low^ cells. It is not clear in our study which CD133 subsets play a role in ATC metastases. Our current xenograft model depends upon the subcutaneous transplantation of CD133 cells into NOD/SCID mice. This location is not a normal niche of thyroid cancer cells and may not faithfully recapitulate the environment experienced by the cancer cells in the original tumor. Although we have shown that tumors derived from either bulk or sorted ARO cell populations are remarkably similar to the primary tumor, it is conceivable that the thyroid bed may better support the growth and differentiation of thyroid CSCs. This point is even more important when we consider that ATC is a locally aggressive type of thyroid tumor with high rate of distant metastases. Therefore, it is critically important to establish an orthotopic model in which tumor cells are injected directly into the thyroid gland [37].

**Figure 7:**
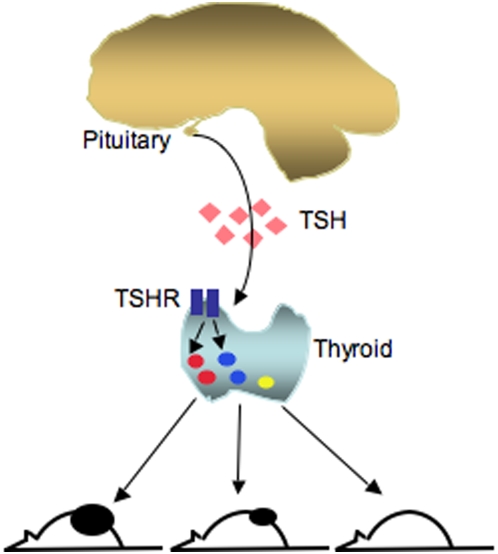
Model depicting the role of TSH signaling in ATC development. TSH, thyroid stimulating hormone, TSHR, the receptor for thyroid stimulating hormone, Red circle, CD133^+/high^ cells, blue circle, CD133^+/low^ cells, yellow circle, CD133^−^ cells.

We found that CD133^+^ cells preferentially overexpress genes, such as Oct4, that are normally enriched in embryonic stem cells. Our previous RT-PCR gene expression profiles indicate that ATC cell lines express the embryonic stem cell markers Rex1, Oct4 and Nanog. The expression of a number of endodermal markers, including Sox17, Foxa2 and Sox 7, is also detected (data not shown). Although these cancer cell lines also express TSHR and the thyroid transcription factor Pax8, other markers of terminally differentiated thyroid cells, such as the sodium-iodide symporter and thyroglobulin, are not expressed in these cell lines (Friedman and Lin, unpublished observations). These results are in agreement with those reported by Zito *et al* who found that ARO/CD133^+^ cells express the thyroblast-specific transcription factor TTF-1 and Oct4, but not thyroglobulin, thyroperoxidase or sodium-iodine symporter [25]. Together these findings indicate that ATC cells have not only begun to proliferate uncontrollably, a property common to all cancer cells, but also that they are losing characteristics of differentiated thyroid cells. Our results reveal a previously unknown link between genes associated with embryonic stem cells and thyroid cells and support the possibility that these genes contribute to the CSC-like phenotypes exhibited by many tumors. These observations suggest that the regulatory networks controlling the function of thyroid cells may also be active in ATCs. Our discovery that TSH can regulate the proliferation and growth of CD133^+^ cells may have important implications. Clinically, the highest incidence of thyroid cancer occurs in patients with abnormally elevated TSH levels. We found that CD133^+^ cells overexpress the TSHR gene. Furthermore, this expression is positively regulated by TSH, implying an autoregulatory feedback mechanism (Fig. 7). It is important to further investigate how TSH affects the proliferation of CD133^+^ cells and, by extension, that of other CSCs. The role of Oct4 activation in cancer survival and the genes and proteins that facilitate the self-renewal of CD133^+^ also warrant investigation.

In summary, our identification of these tumorigenic ATC cells is a critical first step in understanding how TSH signaling affects the growth of CD133^+^ cells and in developing more effective therapies for ATC. The diagnosis of ATC currently relies on a complex panel of pathological parameters and the use of CD133 as a novel marker to identify ATC-cancer-initiating cells may be a more reliable way to detect and monitor cancer progression. These findings should stimulate additional studies to determine whether quantitative or qualitative differences in CD133 expression in ATC have prognostic value. Furthermore, they suggest that CD133 may itself be a potential therapeutic target in this disease.

## Materials and Methods

### Human thyroid cancer cell culture

Two undifferentiated human anaplastic thyroid carcinoma cell lines (ARO and FRO), and two poorly differentiated human papillary thyroid carcinoma cell lines (NPA and TPC), were obtained from Dr. James Fagin (Memorial Sloan-Kettering Cancer Center, New York). ARO, FRO and NPA cell lines were cultured in RPMI-1640 medium supplemented with 10% fetal bovine serum (FBS). The TPC cell line was cultured in Dulbecco's modified Eagle (DMEM, Gibco-BRL) high-glucose medium containing 10% FBS. Cultures were maintained in a humidified chamber in a 5% CO_2_/air mixture at 37°C. For each cell line, 15,000 to 30,000 cells were seeded in chamber slides and cultured until they reached confluence. Cells were harvested using standard methods. Karyotypes were determined for all the human thyroid cancer cell lines using GTG banding. The karyogram data were identical to the previously published reports [38]–[41] and confirmed these cell lines are of human origin.

### Flow cytometry

Cells were dissociated with trypsin/EDTA for three minutes, and then washed and labeled with CD133/1(AC133)-APC antibody (Miltenyi Biotec) and sorted on a MoFlo Cell Sorter. Viability dye was used to eliminate dead cells. Side and forward scatter profiles were used to eliminate cell doublets. Cell purity was evaluated after sorting by FACS analysis. Flow cytometric analysis was performed on a FACS Calibur flow cytometer (BD Biosciences). FACS data were generated using FlowJo software (FlowJo LLC. Ashland, OR).

### Subcutaneous transplantation of bulk and sorted cancer cells into NOD/SCID mice

NOD.CB17-*Prkdc^scid^*/J (NOD/SCID) mice were obtained from the Jackson Laboratory and maintained under specific pathogen-free conditions with the approval of the Institutional Animal Care and Use Committee of Mount Sinai School of Medicine. Bulk and sorted CD133^+/high^, CD133^+/low^ and CD133^−^ cells were re-suspended in PBS/Matrigel and injected subcutaneously into NOD/SCID mice. Mice were sacrificed after four to eight weeks and the tumors were removed, fixed in 10% formalin and embedded in paraffin in preparation for immunohistochemistry. All animal experiments were performed according to approved institutional protocols.

### Alamar blue assay for cell proliferation

Bulk and sorted CD133^+/high^, CD133^+/low^ and CD133^−^ cells were seeded in triplicate into 96-well plates at a concentration of 10^4^ cells/well in RPMI/5% FBS and placed in a humidified chamber of 5% CO_2_/air mixture at 37°C. For cell proliferation assays, Alamar Blue dye (Invitrogen) was added directly into culture media to a final concentration of 10%. After an additional hour of incubation, the plates were read in a fluorescent plate reader. As a negative control, Alamar blue was added to cell-free medium.

### Immunohistochemistry

Immunohistochemistry was performed on 10 archival routinely processed formalin-fixed, paraffin-embedded ATC tissue specimens. Paraffin sections were de-paraffined and then rehydrated with distilled water. The slides were subsequently incubated with anti-human CD133/1 (Abcam Inc.) antibody and visualized with Elite vector Stain ABC systems (Vector Laboratories) and DAB substrate (DakoCytomation) and counterstained with hematoxylin.

### qRT-PCR

Total RNA was isolated with the RNeasy kit (Qiagen) and treated with RNase-free DNase (Qiagen). Two micrograms of total RNA were reverse transcribed into cDNA using the Thermoscript First Strand Synthesis System (Invitrogen). Qiagen QuantiFast SYBR Green PCR kit was used for the qRT-PCR reaction. Human GAPDH was used as the housekeeping gene during the amplifications. The primers used in this study were as follows: *TSHR* (forward), 5′-CCTGAGAATTGTGGTGTGGTTCGTTAG-3′; *TSHR* (reverse), 5′-AGTTTGTAGTGGCTGGTGAGGAGAA-3′; **Oct4** *(forward), 5′-ATGCATTCAAACTGAGGTGCCTGC-3′;* *Oct4* (reverse), 5′-CCACCCTTTGTGTTCCCAATTCCT-3′; *Foxa2* (forward), 5′-GCATTCCCAATCTTGACACGGTGA-3′; *Foxa2* (*reverse*), 5′-GCCCTTGCAGCCAGAATACACATT-3′; *CXCR4* (forward), 5′-AGGGAACTGAACATTCCAGAGCGT-3′; *CXCR4* (reverse), 5′-AAACGTTCCACGGGAATGGAGAGA-3′; *GAPDH* (forward), 5′-TGCACCACCAACTGCTTAGC-3′; *GAPDH* (reverse), 5′-GGCATGGACTGTGGTCATGAG-3′.

### Statistical analysis

Statistical analysis was performed with Prism software. Numerical data are expressed as mean±s.e.m. Statistical differences are considered significant at *P*<0.05.
